# A multiomics approach to identify host-microbe alterations associated with infection severity in diabetic foot infections: a pilot study

**DOI:** 10.1038/s41522-021-00202-x

**Published:** 2021-03-22

**Authors:** Michael Radzieta, Fatemah Sadeghpour-Heravi, Timothy J. Peters, Honghua Hu, Karen Vickery, Thomas Jeffries, Hugh G. Dickson, Saskia Schwarzer, Slade O. Jensen, Matthew Malone

**Affiliations:** 1 South Western Sydney Limb Preservation and Wound Research, South Western Sydney LHD, Sydney, Australia; 2 Infectious Diseases and Microbiology, School of Medicine, Western Sydney University, Sydney, Australia; 3 Surgical Infection Research Group, Faculty of Medicine and Health Sciences, Macquarie University, Sydney, Australia; 4 Immunogenomics Laboratory, Immunology Division, Garvan Institute of Medical Research, Sydney, Australia; 5 School of Science and Health, Western Sydney University, Sydney, Australia; 6 South Western Clinical School, University of New South Wales, Sydney, Australia; 7 Antibiotic Resistance and Mobile Elements Group, Ingham Institute of Applied Medical Research, Sydney, Australia

**Keywords:** Metagenomics, Microbial genetics, Microbiome, Bacteriology, Clinical microbiology

## Abstract

Diabetic foot infections (DFIs) are a major cause of hospitalization and can lead to lower extremity amputation. In this pilot study, we used a multiomics approach to explore the host–microbe complex within DFIs. We observed minimal differences in the overall microbial composition between PEDIS infection severities, however *Staphylococcus aureus* and *Streptococcus* genera were abundant and highly active in most mild to moderate DFIs. Further, we identified the significant enrichment of several virulence factors associated with infection pathogenicity belonging to both *Staphylococcus aureus* and *Streptococcus*. In severe DFIs, patients demonstrated a greater microbial diversity and differential gene expression demonstrated the enrichment of multispecies virulence genes suggestive of a complex polymicrobial infection. The host response in patients with severe DFIs was also significantly different as compared to mild to moderate DFIs. This was attributed to the enrichment of host genes associated with inflammation, acute phase response, cell stress and broad immune-related responses, while those associated with wound healing and myogenesis were significantly depleted.

## Introduction

As an organ, the skin is a formidable barrier to infection, with the epidermis and dermis, populated by a variety of cell types that together form an orchestrated defense against invading pathogens. In the feet of people with diabetes, breaks in the protective barrier of the skin are common and attributed to factors that include peripheral neuropathy, altered foot architecture, peripheral arterial disease, trauma, and altered immune responses^1^. It is for these reasons why infections of the skin, soft tissue and bone, in the feet of people with diabetes are predominant causes of hospitalization and lower extremity amputation^2^.

The diagnosis of diabetic foot infections (DFIs) and the classification of severity is a pivotal juncture for clinicians. Expert guidelines such as those by the International Working Group for the Diabetic Foot (IWGDF) support clinicians to navigate this challenging pathology^3^. The diagnosis of DFI and its severity are primarily based on clinical observations (with adjunctive imaging and laboratory tests), and are defined clinically as the presence of manifestations of an inflammatory process in any tissue below the malleoli in a person with diabetes mellitus^3^. Most DFIs will initially involve the superficial skin, however microorganisms can spread contiguously to involve deeper structures. This may progress to invoke systemic symptoms (e.g., febrile, nausea, and vomiting), marked leucocytosis or major metabolic disturbances. Whilst severe infections are less common in patients with a DFI, their presence may imply a potentially limb threatening (or even life-threatening) infection^2^. To add further to the complexity of this challenging pathology, observations have implicated several ill-defined immunological disturbances^4,5^, masked infective symptoms^6^, and other co-morbidities^2^ as attenuating the problem. Managing DFIs and understanding the pathogenesis is therefore of paramount importance.

Research to date on the bacteriological composition of DFIs has predominantly utilized conventional culture-dependent methods. However, culture-independent approaches based on molecular techniques have the potential to provide new perspectives and are increasingly being adopted. For example, several research groups have employed multiplex PCR assays to identify specific virulence determinants of the common DFI pathogen *Staphylococcus aureus*, independent to sequencing entire microbial genomes^7–9^. We have previously used culture-independent, amplicon-based sequencing methods (i.e., bacterial 16S rRNA gene sequencing) to explore the microbiome of tissues from infected diabetic foot ulcers (DFUs)^10^. This approach has provided an extended view of the DFI microbiome, however the interpretation of these findings (reporting to genus level identification) and their relevance to clinical care remains largely ambiguous. Importantly, several limitations of short-read amplicon sequencing and the targeting of only taxonomic genes of interest, have restricted insights into many facets of the host–microbe infective process. Recently, Kalan and colleagues have circumvented the limitations of 16S rRNA gene sequencing by employing shotgun metagenomics of non-infected DFUs^11^. The study provided an intriguing insight into several facets of microbial taxonomic and genetic markers associated with potential function and clinical outcomes.

In the present pilot study, tissue punch biopsies were obtained from 36 patients with DFI presenting to a high-risk foot service. Tissue specimens were analysed by shotgun metagenomic sequencing (Metagenome) and dual RNA-seq (Metatranscriptome) analyses to (i) Investigate the diversity, community composition and functional potential of microbial communities in DFIs, (ii) determine the relative activity of microbial communities in DFIs, and (iii) identify differentially expressed host–microbial genes present during DFIs.

## Results

### Community composition and functional analysis of microbiomes associated with DFIs

**Fig. 1:**
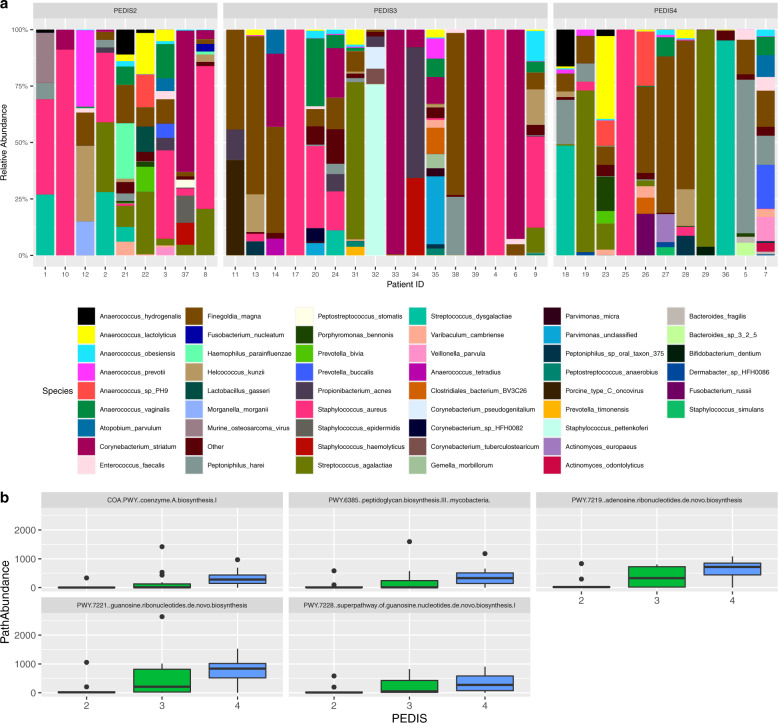
Analysis of metagenomic shotgun sequencing data using Humann2. **a** Bar chart representing the 30 most highly abundant species (%) for 36 individuals with DFIs grouped by infection severity (PEDIS). Each bar represents individual species level identification. **b** Boxplots identifying metabolic pathways which are significantly different in abundance between infection severity groups (LDA score >2 (*p* < 0.05)).

The microbial community composition and potential microbial function of 36 patients with varying severities of DFI (Mild—PEDIS 2 = 9, Moderate—PEDIS 3 = 15, Severe—PEDIS 4 = 12) was examined using shotgun metagenomic sequencing (Data 1). We obtained a median of 13,230,546 reads per sample with a median of 744,700 reads mapping to the ChocoPhlAn database using Humann2 (range = 1765 to 11,273,346 reads) (Supplementary data 1). Filtered reads were analysed using the Humann2 pipeline, employing the ChocoPhlAn and Uniref90 databases for taxonomic and functional classification of microbial reads, respectively^12^. Bacterial relative abundance was assessed at the species level, which identified several highly abundant organisms including; *Staphylococcus* (20.8%) (*S. aureus*, 83%), *Streptococcus* (16.6%) (*S. agalactiae*, 65.6%, *S. dysgalactiae*, 34.3%), *Finegoldia* (15.2%) (*F. magna*, 100%), *Corynebacterium* (12.9%), (*C. striatum*, 95%) and *Anaerococcus* (9.6%) (*A. vaginalis*, 26%, *A. lactolyticus*, 24%, *A. prevotii*, 14.6%, *Anaerococcus*, 14.5%, *A. hydrogenalis*, 10.4%, *A obesiensis*, 8.3%, *A tetradius*, 2.2%) across all samples (Fig. 1a and Supplementary data 2). PCA analysis utilizing the relative abundance data did not identify any clustering of specific PEDIS infection severities, thus demonstrating no difference in microbial composition based on the severity of infection (Supplementary Fig. 1). Analysis using the linear discriminant analysis effect size (LEfSe) test was then used to identify any bacterial taxa, which may characterize key differences between each PEDIS infection severity. LEfSe analysis of taxa based on relative abundance identified the increased presence of *S. agalactiae* within PEDIS four patients (LDA Score (Log10) = 5.17, *p* = 0.03).

In addition to taxonomic analysis, shotgun metagenomics enabled functional analysis of potential microbial pathways using MetaCyc pathway definitions^13^. Analysis was focused on using the “Pathabundance” and “Genefamily” outputs from humann2 (Supplementary data 3 and 4), which were merged and normalized (LogCPM) prior to regrouping into KEGG functional categories and analysis using LEfSe. Analysis of the Pathabundance output was utilized to examine metabolic differences in the microbiome associated with each PEDIS infection severity, with five pathways being identified as over-represented within PEDIS 4 infections. All of these pathways were associated with growth or nucleotide metabolism (Fig. 1b). Next we examined differences in genefamily abundances between each PEDIS infection severity. LEfSe analysis identified three genes that encode ABC transporter proteins over-represented within PEDIS 4 infections including K06147 (ATP-binding cassette, subfamily B), K01990 (ABC-2 type transport system ATB-binding protein) and K16785 (energy-coupling factor transport system permease protein EcfT) (*p* < 0.05).

### Metabolically active microbial communities within DFI

**Fig. 2:**
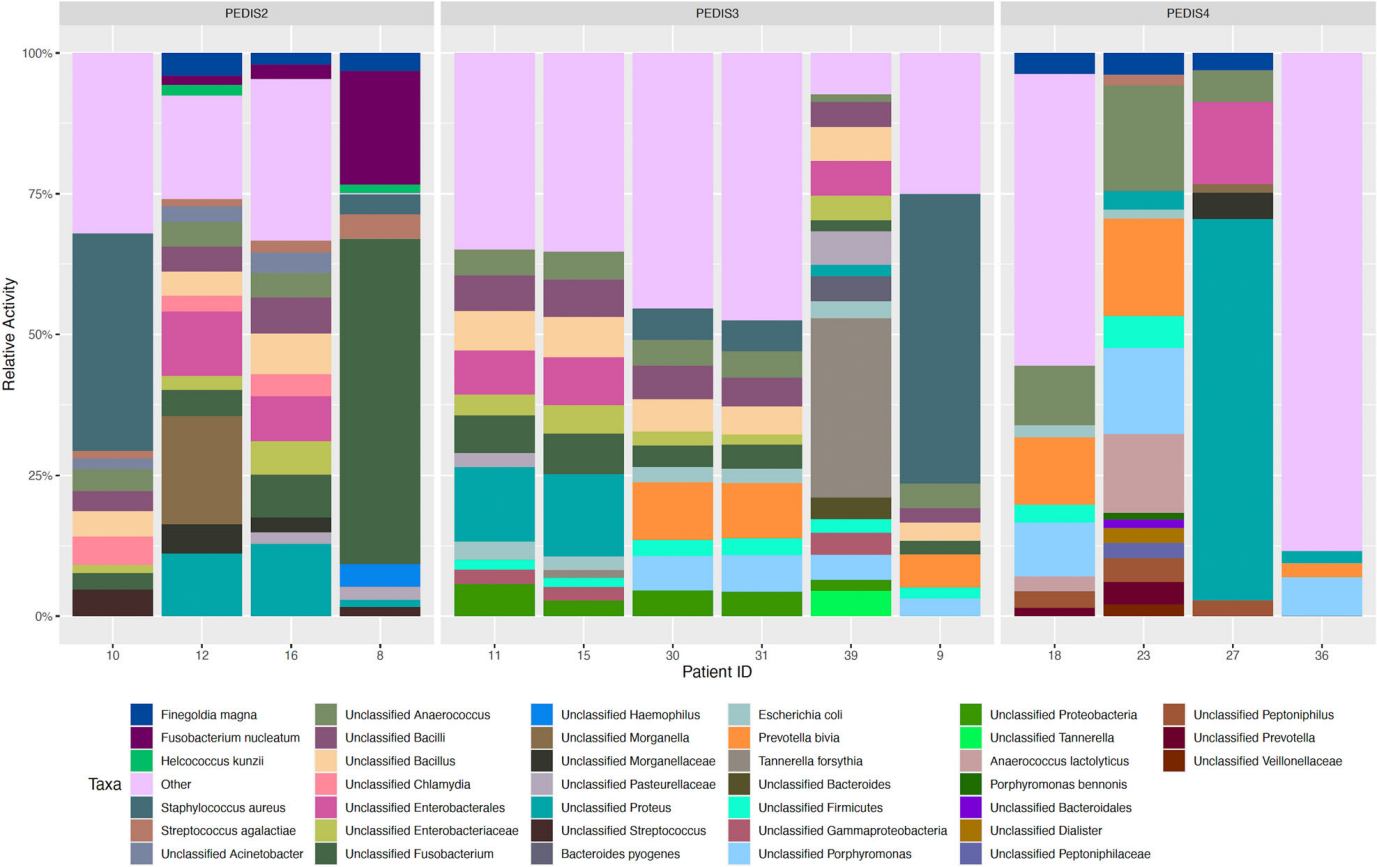
Bar chart showing the top 20 most metabolically active taxa across each sample following metratranscriptomic analysis with the SqueezeMeta pipeline. Activity was based on normalized read counts (LogCpm) annotated to individual taxa and grouped according to infection severity (PEDIS group). Only taxa which comprises >1% of the total annotated microbiome are shown.

Metatranscriptome datasets were used to identify metabolically active microbial communities in DFI using the SqueezeMeta pipeline, with a median of 25,583,464 reads being used as input for SqueezeMeta (Supplementary Data 2). Taxonomic assignments are reported at multiple levels to illuminate species as well as unclassified higher taxa which represent >1% of the total microbial composition, and comparisons were made across PEDIS infection severities to identify relative microbial activity. A PCA analysis was performed as a first step and identified variation across PEDIS infection severities, with samples not generally clustering according to infection severity (Supplementary Fig. 2). In PEDIS 2 and PEDIS 3 infections, the most active taxa are *S. aureus* (10%), *Fusobacterium* (10%), *Bacillus* (5%), *Anaerococcus* (4%), *Enterobacteriacae* (3%), *Streptococcus agalactiae* (2%), *Proteobacteria* (2%), *Morganella* (2%), and *Porphyromonas* (2%) (Fig. 2 and Supplementary data 6). PEDIS 4 infections were greater in heterogeneity, with the most active taxa being *Proteus* sp. (18%), *Anaerococcus* (9%), *Prevotella bivia* (8%), *Porphyromonas* (8%), *Anaerococcus lactolyticus* (4%), and *Enterobactarales* (4%). Across all samples, a varying proportion of reads mapping to “others” were from taxa contributing <1% of total reads per individual was observed (29.5%, ±24). These included reads from genera with few transcripts and likely false positives, as well as reads associated with samples with a more complex and diverse community. In PEDIS 2 and PEDIS 3 infections, reads mapping to “Others” accounted for 27.4% (±15.2) of total reads, and this number was greater in PEDIS 4 infections due primarily to patients 18 and 36 (34.7%, ±42.8).

### Differences in the microbial transcriptomes of PEDIS infection severities

Functional analysis of the microbial transcriptomes was carried out using data which was generated from annotating reads to the KEGG database (Supplementary data 7 and 8). This allowed for the interrogation of any changes at a global and targeted level. In order to establish if any variations in the functional profiles of PEDIS infection severities existed, a PCA analysis was performed on normalized read counts annotated from KEGG (Supplementary Fig. 3). This revealed that the PEDIS 2 and 3 patients generally grouped together, with the exceptions being samples 9, 11, and 15, which have similar functional profiles to the PEDIS 4 patients.

**Fig. 3:**
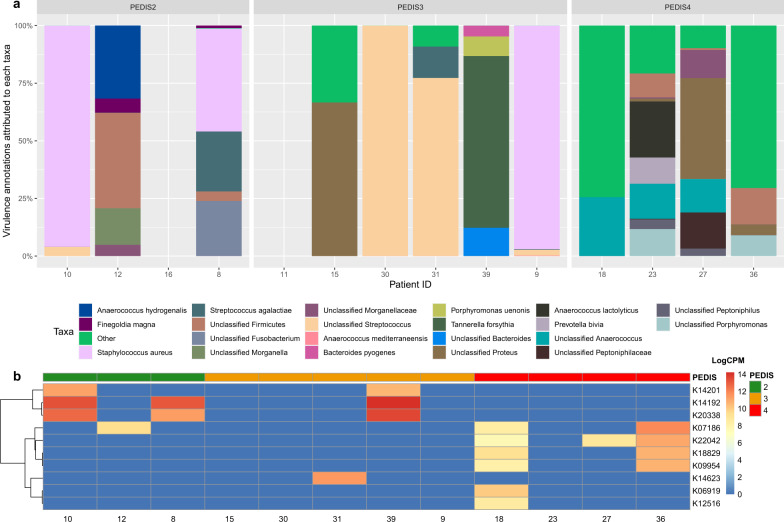
Functional analysis of annotations isolated by the keyword “virulence”. **a** Bar chart showing the top 10 taxa by with virulence annotations. Annotations were extracted using the “subsetTax” tool included with the SQMtools R package. Patients 16 and 11 had no annotations related to “virulence” within these samples. **b** Heat map of the top ten most enriched virulence proteins across all PEDIS infection severities. Read counts were normalized prior to applying a multilevel analysis (ANOVA) fitting all contrasts between groups to identify the top ten enriched genes. Patients are grouped by infection severity (PEDIS) with features clustered using Ward clustering.

In the context of DFIs, we focused on functional annotations relating to virulence that were identified from corresponding taxa across the different PEDIS infection severities. In total, a median of 94 virulence annotations were extrapolated from the PEDIS 2 and 3 Infections, compared to a median of 32,206 annotations from the PEDIS 4 infections. Taxonomic analysis revealed that aerobic Gram-positive cocci were the main producers of virulence factors within PEDIS 2 and 3 infections, with the most prominent taxa being *S. aureus* and *Streptococcus*. Conversely, there was a greater diversity of taxa producing proteins associated with virulence within PEDIS 4 infections, with a large proportion being anaerobic or facultative anaerobic clades such as *Anaerococcus*, *Porphyromonas*, and *Proteus*. (Fig. 3a and Supplementary Data 7). A multilevel analysis (ANOVA) (i.e., fitting all contrasts PEDIS 2vs3, 2vs4, 3vs4) was performed to identify differences in virulence factors expressed across infection severities (Fig. 3b). Several factors were identified as being more prominent within PEDIS 2 and 3 infections, including K14192 (clumping factor B) and K20338 (MarR transcriptional regulator), both of which are virulence factors associated with *S. aureus* infections. Within PEDIS 4 infections, virulence factors identified were associated to a wider diversity of bacteria, including K07186 (SMP membrane protein), K22042 (hlyU transcriptional regulator), K18829 (antitoxin VapB), and K09954 (YpeB).

We next performed a closer analysis of between group variations (2vs3, 3vs4) in gene expression, with genes being considered differentially expressed if there was a >log2 fold-change in transcripts (FWER < 0.05). Between PEDIS 2 and PEDIS 3 infections, no genes associated with virulence were identified to be differentially expressed. Conversely, when comparing PEDIS 4 to each group (2vs4, 3vs4) two genes were identified as enriched within the PEDIS 2 and 3 infections relative to PEDIS 4, including K14192 (FC = −14.21) and K20338 (FC = −13.26) (Table 1, Supplementary Fig. 4, and Supplementary data 8).

**Table 1:** Summary of the top enriched or depleted microbial virulence DEGs in PEDIS 4 compared to PEDIS 3 diabetic foot infections (logFC, FWER < 0.05).

KEGG No.	Protein	Gene	LogFC	FWER
*PEDIS 3 enriched virulence DEGs*				
K14192	Clumping Factor B	*clfB*	−14.21	0.015
K20338	MarR family transcriptional regulator	*rot*	−13.26	0.036

### Differences in the host transcriptomes of PEDIS infection severities

We used edgeR^14^ to examine differential expression of replicated count data between infection severities. Normalized read counts (LogCPM) were used to perform principal coordinate analysis (PCA) to demonstrate variation among datasets (Supplementary Data 9 and Supplementary Fig. 5). Examination of the plots revealed some interesting features. Firstly, we identified that PEDIS 2 and PEDIS 3 infection transcriptomes were generally indistinct from each other and clustered together. Secondly, PEDIS 4 transcriptomes demonstrated a greater heterogeneity in their gene expression profiles and were generally well-separated from PEDIS 2 and PEDIS 3, indicating that PEDIS 4 infections in this dataset had distinct differences in host gene expression patterns.

**Fig. 4:**
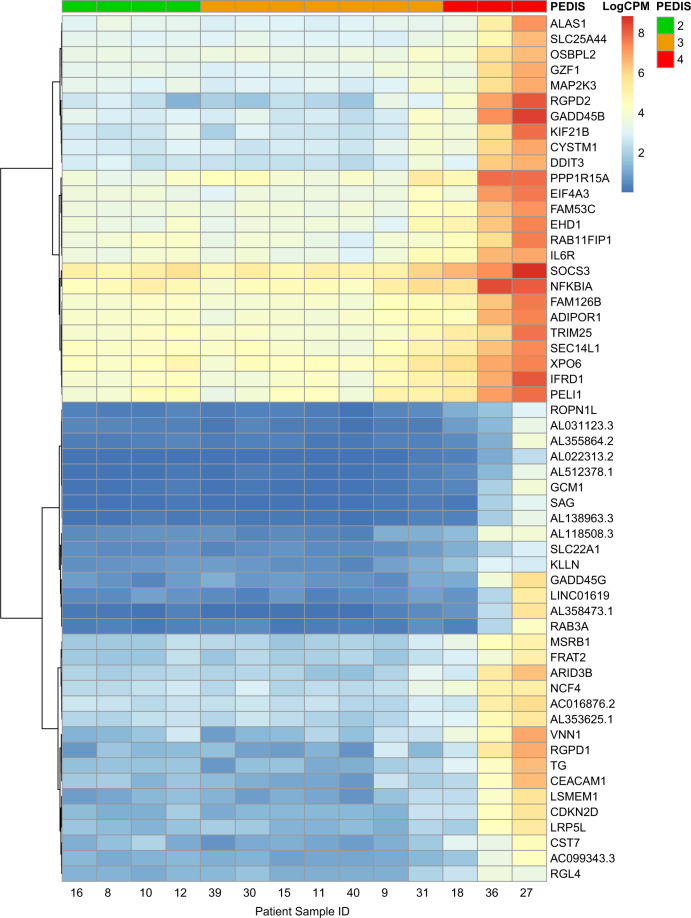
Heat map of the top 50 most enriched host genes across all PEDIS infection severities. Read counts were normalized prior to applying a multi-level analysis (ANOVA) fitting all contrasts between groups to identify the top 50 enriched genes. Patients are grouped by infection severity (PEDIS) with features clustered using Ward clustering.

We next sought to investigate if any host driven DEGs are evident between PEDIS infection severities. A multilevel analysis fitting all contrasts was performed on normalized read counts to identify the top 50 enriched host genes differentially expressed across all three PEDIS infection severities (Fig. 4). Both PEDIS 2 and PEDIS 3 infections are indistinguishable from a gene expression profile perspective, and testing yielded no statistically significant differential expression. These results suggest that there are no or limited differences in the host gene expression between PEDIS 2 and PEDIS 3 infections. Conversely, a unique expression profile is evident within PEDIS 4 infections (predominantly patients 27 and 36). Interestingly, several genes associated with the regulation of the immune response are highly enriched within patients 36 and 30, including SOCS3, NFKBIA GADD45B, RGPD2, and RGPD1.

Further exploration of between group variations was undertaken with genes being considered differentially expressed if the FWER was <0.05. In keeping with previous trends noted in the PCA plot and heatmap, there was no significant difference in DEGs between PEDIS 2 and PEDIS 3 infections. In contrast, analysis of DEGs between PEDIS 3 and PEDIS 4 infections identified highly enriched genes within PEDIS 4 infections (Table 2 and Supplementary Fig. 6). These highly enriched genes were most commonly associated with the host immune response and included RGBD2, GADD45B and GADD45G.

**Table 2:** Top 20 enriched host DEGs in PEDIS 4 compared to PEDIS 2–3 diabetic foot infections. Values in log fold change (LogFC) column indicate fold change ranked from highest to lowest (FWER < 0.05).

Gene	Description	Function/GO biological process	logFC	FWER
RGPD2	RANBP2-like and grip domain-containing protein 2	GTP-binding protein of the RAS superfamily that is associated with the nuclear membrane and is thought to control a variety of cellular functions through its interactions with other proteins	7.0	0.00
TG	Thyroglobulin	Primary functions include iodide storage and thyroid hormonogenesis. Other gene ontology processes include regulation of signaling receptor activity, and signal transduction	5.9	0.01
AC099343.3	Novel transcript	Unknown	4.1	0.01
CST7	Cystatin 7	Role in immune regulation through inhibition of cysteine peptidases which effect varying immune cells (natural killer cells and Eosinophils)	4.3	0.01
FAM53C	Family with Sequence Similarity 53 Member C	Function unknown	3.9	0.01
GADD45B	Growth arrest and DNA damage-inducible protein GADD45 Beta	Levels are increased following stressful growth arrest conditions and treatment with DNA-damaging agents. The genes in this group respond to environmental stresses by mediating activation of the p38/JNK pathway via their proteins binding and activating MTK1/MEKK4 kinase, which is an upstream activator of both p38 and JNK MAPKs. The function of these genes or their protein products is involved in the regulation of growth and apoptosis	6.5	0.01
GADD45G	Growth arrest and DNA-damage-inducible protein GADD45 gamma		6.5	0.01
AL031123.3	Novel transcript	Function unknown	5.4	0.02
MSRB1	Methionine sulfoxide reductase B1	Plays a protective role against oxidative stress by restoring activity to proteins that have been inactivated by methionine oxidation	3.4	0.02
EIF4A3	Eukaryotic translation initiation factor 4A3	Protein coding gene belongs to the Asp-Glu-Ala-Asp (DEAD) box superfamily. Mostly unknown transcriptome wide functions but limited data to identify a role in cellular functions, such as cell cycle control	4.9	0.02
ROPN1L	Rhophilin associated tail protein 1 like	Involved in fibrous sheath integrity and sperm motility, plays a role in PKA-dependent signaling processes required for spermatozoa capacitation	4.5	0.02
ALAS1	Delta-aminolevulinate synthase 1	Involved in heme biosynthesis via negative feedback-mediated regulation	4.6	0.03
AC016876.2	Novel transcript	Function unknown	4.0	0.03
TRIM25	Tripartite motif containing 25	Member of the tripartite motif (TRIM) family and TRIM25 has involvement in pathways for Interferon gamma signaling and cytokine signaling in immune system	3.8	0.03
FAM126B	Family with sequence similarity 126 member B	Function unknown	3.8	0.03
RAB11FIP1	RAB11 family interacting protein 1	A Rab11 effector protein involved in the endosomal recycling process. Also involved in controlling membrane trafficking along the phagocytic pathway and in phagocytosis	4.4	0.03
ARID3B	AT-rich interaction domain 3B	Function unknown	5.0	0.04
CDKN2D	Cyclin dependent kinase inhibitor 2D	Member of the INK4 family of cyclin-dependent kinase inhibitors. Prevents the activation of the CDK kinases, thus functions as a cell growth regulator that controls cell cycle	4.8	0.04
LRP5L	Low density lipoprotein receptor-related protein 5 like	Function unknown	4.5	0.04
IFRD1	Interferon-related developmental regulator 1	Gene coding protein expressed by mature neutrophils, modulating cell differentiation and increasing oxidative stress and pro-inflammatory cytokines	4.3	0.04
XPO6	Exportin 6	Mediates the nuclear export of actin and profilin-actin complexes in somatic cells	3.3	0.04

Given that previous statistical testing identified no significant difference in DEGs between PEDIS 2 and PEDIS 3 infections, a Gene Set Enrichment Analysis (GSEA) was performed to explore any differences between PEDIS 3 and PEDIS 4 infections. The ranked gene list was based on three ontologies; Hallmark gene expression, Gene Ontology (GO), and curated gene sets from the Broad Institute. As a consequence of increasing PEDIS infection severity, significant alterations in the host transcriptome were evident in patients with PEDIS 4 infection, with many of the highly enriched genes being associated with inflammatory responses, immune-related responses, acute-phase responses, and cell stress responses, while those associated with wound healing and myogenesis were significantly depleted (Table 3).

**Table 3:** Summary of the most significant hallmark host responses in patients with PEDIS 4 when compared to PEDIS 3.

NAME	Process category	Description	NES	FWER
*Enriched host pathways in PEDIS 4*				
TNFA_SIGNALING_VIA_NFKB (TNF-alpha/nuclear factor kappa-light-chain-enhancer of activated B cells)	Signaling	Genes regulated by NF-kB in response to TNF	1.9023656	0
IL6_JAK_STAT3_SIGNALING (Interleukin 6/Janus kinase/signal transducers and activators for transcription	Signaling	Genes upregulated by IL6 via STAT3 e.g., during acute phase response	1.7342762	0
INFLAMMATORY_RESPONSE	Immune	Genes defining inflammatory response	1.6779492	0
PI3K_AKT_MTOR_SIGNALING (Phosphatidylinositide 3 kinases/Protein kinase B/mammalian target of rapamycin.	Signaling	Genes upregulated by activation of the PI3K/AKT/mTOR pathway, regulating cell proliferation, survival, and metabolism	1.6093148	0.00
HYPOXIA	Pathway	Genes upregulated in response to low oxygen levels	1.5994961	0.00
REACTIVE_OXIGEN_SPECIES	Pathway	Genes upregulated by reactive oxygen species (ROS)	1.578071	0.00
UV_RESPONSE	DNA damage	Genes upregulated in response to ultraviolet (UV) radiation	1.5617301	0.01
APOPTOSIS	Pathway	Genes mediating programmed cell death by activation of caspases	1.5393971	0.01
IL2_STAT5_SIGNALING (Interleukin 2/signal transducers and activators for transcription)	Signaling	Genes upregulated by STAT5 in response to IL2 stimulation	1.5364993	0.02
HEME_METABOLISM	Metabolic	Genes involved in metabolism of heme (a cofactor consisting of iron and porphyrin) and erythroblast differentiation	1.534089	0.02
HALLMARK_P53_PATHWAY (Tumor protein 53)	Proliferation	Genes involved in p53 pathways and networks in response to stress signals that impact upon cellular homeostatic mechanisms that monitor DNA replication, chromosome segregation, and cell division	1.5275651	0.02
ALLOGRAFT_REJECTION	Immune	Genes upregulated during transplant rejection/ alloimmune response to non-self-antigens expressed by donor tissues	1.5268086	0.02
MTORC1_SIGNALING (Mammalian target of rapamycin complex 1)	Signaling	Genes upregulated through activation of mTORC1 complex	1.5246518	0.02
***Depleted host Pathways in PEDIS 4***				
NAME	Process category	Description	NES	FWER *p*-val
EPITHELIAL_MESENCHYMAL_TRANSITION	development	Genes defining epithelial–mesenchymal transition, as in wound healing, fibrosis, and metastasis	−1.9640744	0
MYOGENESIS	development	Genes involved in development of skeletal muscle (myogenesis)	−1.6926087	0

The dataset was interrogated for pathway enrichment or depletion using the canonical pathways from the MsigDb collection using Gene Set Enrichment Analysis (GSEA). The GSEA pathway analysis results show host gene enrichment involving inflammatory, immune and signaling activation, and host gene depletion involving wound repair. NES: normalized enrichment score; FWER: family-wise error rate. Values in log fold change (logFC) column indicate fold change ranked from highest to lowest (*p* < 0.05, FWER < 0.05).

### Correlation of clinical variables with host gene expression

Diabetic foot infection and its severity is a clinical diagnosis based on the presence or absence of signs and symptoms of inflammation, with laboratory and imaging studies providing adjunctive evidence. We sought to correlate the clinical variables; duration of the DFU at presentation, White cell count [WCC], erythrocyte sedimentation rate [ESR], C-reactive protein [CRP], neutrophil count and glycosylated hemoglobin [HbA1C], against the host transcriptome in fourteen individuals. Alterations in host DEGs against clinical variables were identified as being confined to singular individuals, with no significant trends between host DEGs evident across all individuals. One exception to this was the clinical variable WCC, where all individuals exhibited an enrichment of Egr-1 (early growth response gene-1) (FWER = 0.04). Egr-1 further demonstrated increased enrichment in a linear response to an increasing white cell count (Supplementary Fig. 7).

## Discussion

To define the community composition and molecular determinants (potential function and activity) of the host–microbe interaction at the site DFIs, we performed shotgun metagenomic sequencing and total RNA-Seq of tissue punch biopsies from infected diabetes foot ulcers. Clinical and laboratory data from individuals with DFIs were collected to classify the severity of infections, and these were subsequently used to compare genomic datasets. Shotgun metagenomic datasets were utilized for taxonomic classifications, with the advantage of achieving species-level resolution of identified taxa. We observed many previously reported microorganisms-pathogen/s associated with DFI, with the highest relative abundances aligning to aerobic gram-positive cocci (*Staphylococcus aureus*, *Streptococcus agalactiae*, and *Streptpcoccus dysgalactiae*), followed by *Corynebacterium striatum* and species belonging to members of the class clostridia (*Anaerococcus*, *Finegoldia magna*, *Helococcus kunzii*).

A relative abundance bar chart was constructed from raw reads with patients stratified by infection severity. At the individual patient level, there is marked heterogeneity in microbial composition, however, PCA analysis of the combined dataset does not reveal any differences in the overall microbial composition between PEDIS infection severities. To further elucidate any potential individual taxa which would characterize any key differences between PEDIS infection severities, an LDA effect size (LEfSe) test was performed. This identified *S. agalactiae* and *S. dysgalactiae* as being over-represented within PEDIS 4 infections. These findings correlate with previous publications that have identified aerobic Gram-positive cocci as primary drivers associated with DFIs^10,15,16^.

We next aimed to analyse the functional profile of the metagenomic dataset using a curated database of experimentally elucidated proteins (KEGG). This revealed several over-represented gene families within PEDIS 4 infections. In particular, ABC transporter proteins accounted for a large proportion of over-represented pathways/proteins, which can be utilized by pathogens as mechanisms to acquire essential nutrients from the host while mediating the effects of toxicity. We identified; K06147 (ATP-binding cassette, subfamily B), K01990 (ABC-2 type transport system ATB-binding protein), K16785 (energy-coupling factor transport system permease protein EcfT), YadG (putative ABC transporter ATP-binding protein); EcfT and YadG are type II and type III transporters with predicted functions in antibiotic export and broad virulence functions through the acquisition of transition metals, peptides and amino acids, respectively^17^.

Analysis of differentially abundant microbial pathways identified those primarily involved in growth and metabolism, notably MetaCYC pathways 7221, 7228, and 7219, which are involved in the synthesis of nucleotide precursors. The over-representation of these pathways within PEDIS 4 infections may be indicative of a greater microbial diversity within these wounds, given that changes in the microbiota can significantly alter the microbial functional profile^18^.

Next we performed an analysis of metatranscriptome datasets to provide a unique insight into the contribution of metabolically active microorganisms in DFI, with the top five most active taxa being *S. aureus*, *Proteus, Anaerococcus*, *Fusobacterium*, and *Bacillus*.

The observations of *Bacillus* as being metabolically active within DFIs is an interesting finding given their rarity in published reports of DFI^19^. Conventional microbiology would assume that under a steady-state growth, most active bacteria would also be the most abundant, with higher growth rates resulting in greater biomass^20^. Using these assumptions, it is also expected that uncommon or rare bacteria are slow growing and may only become abundant under selective environmental conditions^21^. Molecular techniques primarily employed in environmental research has illustrated that rare bacteria may be disproportionately active relative to their abundances, and that low-abundance taxa may contribute disproportionately to ecological and biogeochemical processes relative to their abundances^22,23^.

Further analysis of the metatranscriptome dataset identified several predominant Staphylococcal virulence factors enriched within PEDIS 2 (mild) and 3 (moderate) DFIs, including clumping factor B and the MarR transcriptional regulator^24–27^. The relative activity of *S. aureus* within microbial communities by differential expression analysis suggests that *S. aureus* likely plays a significant role in the pathogenicity of mild (PEDIS 2) to moderate (PEDIS 3) infections. This data provides further support for current DFI guidelines advocating for antibiotic regimens to provide coverage towards aerobic Gram-positive cocci for non-severe DFIs^3^. Conversely, virulence factors enriched within PEDIS 4 patients were generally associated to multiple species, including SMP (*E. coli*), hlyU (*Vibrio*), VapB (*Rhodococcus*), and YpeB (*Bacillus*)^28–31^.

To investigate the host response during microbial infection, gene expression profiles were calculated by the differential expression analysis package edgeR, and genes showing statistically significant changes in their expression level were explored through a multilevel ANOVA model. We identified that host transcriptomes in PEDIS 2 and PEDIS 3 were similar, however host transcriptomes in PEDIS 4 infections demonstrated distinct differences in gene expression patterns. As a consequence of increasing infection severity significant alterations in the host transcriptome were evident, with many of the highly induced genes being associated with (i) inflammatory—signaling responses, (ii) acute-phase responses, (iii) immune-related responses, and (iv) cell stress responses. Because inflammation is a hallmark of DFI, it was expected that various transcripts for host immunomodulatory proteins would be strongly enriched.

We further sought to explore host–microbe interactions which may illustrate why some patients develop greater clinical and systemic features of infection. Broadly assessing microbial composition (active and inactive bacteria) as a potential explanation did not yield any clear insights. We generally observed that in comparison to mild and moderate DFIs, severe DFIs are greater in diversity containing both aerobic Gram-positive cocci and anaerobes (polymicrobial). Analysis of microbial function, however, reveals severe DFIs are associated with multispecies virulence factors whereas mild to moderate DFIs are typically associated with *S. aureus* enriched virulence factors. Therefore, one potential explanation of a heightened host immune response may be explained by the presence of multiple infecting microorganisms, their synergism to induce virulence and pathogenicity, alter the infected niche, or modulate the host immune response^30^.

Clinical metadata was collected from each patient to investigate for any correlations against host transcripts. Overall there were no correlations identified with the exception of white cell count. We identified enrichment of Egr-1 (early growth response gene-1) in a linear trend against increasing white cell counts. Previous studies have outlined the role of Egr-1 in the immune response to microbial infections, specifically the role of bacterial adhesions in inducing the expression of Egr-1 from host cells^32^. Egr-1 is broadly expressed in different cell types and is rapidly induced by a wide range of stimuli, including growth factors, cytokines, stress, and injury. Elevated Egr-1 expression has previously been linked to production of inflammatory mediators in pulmonary diseases^33,34^.

Recently, Pang and colleagues^35^ illustrated that Egr-1 plays a detrimental role in host defense against *Pseudomonas aeruginosa* acute lung infection by promoting systemic inflammation and negatively regulating nitric oxide production that assists with bacterial clearance. We observe enrichment of Egr-1 within all patients in this study, a likely reflection of bacterial infection due to cell adhesion and invasion. By analysing the microbial transcriptome of DFI, we identify several adhesion proteins (fimbrial protein A and hemagluttinin protein) highly enriched within PEDIS 4 infections. This may explain the increased expression (and thus enrichment) of Egr-1 in tandem with a heightened immune response observed clinically as a severe (PEDIS 4) infection. Further research in larger datasets identifying trends in Egr-1 between infected and non-infected patients may lead to targets for improving the diagnostic accuracy and management of DFIs.

This work is constrained by several limitations. The study sought to enroll consecutive patients presenting with DFI who had not received any antibiotic therapy within 2 weeks prior to presentation. The choice of sampling method was by tissue punch biopsy, which required sectioning of the biopsy to undertake conventional culture, DNA, and RNA analysis. A limitation to this was the ability to obtain enough tissue material from all patients to carry out all analyses, and this accounts for the lower number of RNA metatranscriptome datasets. The 39 patients enrolled in this study allowed for a preliminary insight into better understanding DFIs, however it is not possible with this sample size to make larger generalizations to the DFI population. Additionally, our analysis was a within-group (patients only with DFIs) methodology. We acknowledge that future studies require control samples (healthy in-tact skin) for comparison, and/or patients stratified by disease status i.e., healing DFU non-infected, chronic DFU non-infected, DFU with chronic infection, and DFU with acute infection.

The disparity between metagenome and metatranscriptome datasets in the most commonly aligned genera/species between the two approaches is not entirely unexpected. A limitation of shotgun metagenomics is the inability to distinguish active from inactive members of a microbiome and RNA-seq circumvents this limitation, as it targets expressed transcripts within a microbiome at a given point^36^. However, even when viewing microbial activity to understand which microorganisms within a community maybe contributing to an infective process, itself needs to be applied with a level of caution. Any intact microbial cells within tissue samples will have some level of metabolic activity to support basic cell maintenance, in addition to any further processes such as virulence, pathogenicity, growth, and repair. Under these circumstances delineating “active” bacteria from “inactive” or “less active” bacteria are not well defined in the literature. Furthermore, most microbes in nature are aggregate communities (biofilms) with altered growth, but a microbe can be “active” and contributing to key processes without growth^37^.

Lastly, analysis of the metagenomic and metatranscriptomic datasets were not completed using the same pipeline and database. Metagenomic analysis relied on the Humann2 pipeline which utilizes the proprietary ChocoPhlAn database derived from genomes available from NCBI, focusing on mapping reads to select marker genes. Alternatively, metatranscriptomic analysis was completed using SqueezeMeta^38^, which utilizes the GenBank and KEGG databases for mapping to entire genomes, furthermore SqueezeMeta also assembles the metatranscriptomes prior to the mapping step. The differences in selected pipelines would explain some of the disparities noted between the two datasets. A direct comparison is therefore not possible, highlighting the difficulties associated with combining metatrancriptomic and metagenomic analysis.

In summary, the results of this pilot study identify aerobic Gram-positive cocci are abundant and highly active in mild to moderate DFIs. This supports current international guidelines^3^ proposing antimicrobial regimens targeting key pathogens that include *S. aureus* and *S. agalctaie* and *S. dysgalctiae*. The results of this study may not apply to patients with DFIs residing in tropical/sub-tropical climates where the prevalence of Gram-negative rods (especially *P. aeruginosa)* appears to be higher than reported in other parts of the world^3^.

In particular, virulence genes belonging to *Staphylococcus aureus* are predominant, whilst multi-species virulence genes are greater in severe DFIs. One potential explanation of a heightened host immune response identified in severe DFIs may be explained by the presence of multiple infecting microorganisms, their synergism to induce virulence and pathogenicity, alter the infected niche, or modulate the host immune response.

## Methods

Key resources box

**Table:** 

Reagent or resource	Source	Identifier
*Biological samples*		
Diabetic foot ulcer specimens (Tissue Biopsies)	Human in vivo	See metadata.csv in supplement for full metadata
*Critical commerical assays*		
QubitTM dsDNA HS Assay Kit	Life Technologies	Cat#Q32854
Zymo host zero microbial DNA kit	Zymo	Cat#D4310
TRIzol plus total transcriptome isolation kit	ThermoFisher	Cat#12183555
*Deposited data*		
Total RNA sequencing data	This paper	Sequence Read Archive (SRA)/NCBI (http://www.ncbi.nlm.nih.gov/sra) accession number PRJNA563930.
Shotgun metagenomic sequencing data	This paper	Sequence Read Archive (SRA)/NCBI (http://www.ncbi.nlm.nih.gov/sra) accession number PRJNA610303.
*Software and algorithms*		
HUMAnN2	Franzosa et al.^12^	http://huttenhower.sph.harvard.edu/humann
Bowtie2	Langmead^46^	http://bowtie-bio.sourceforge.net/bowtie2/index.shtml
BBTools	Bushnell et al.^41^	https://sourceforge.net/projects/bbmap/
Trim Galore	Marcel^42^	https://github.com/FelixKrueger/TrimGalore
STAR	Dobin et al.^43^	https://github.com/alexdobin/STAR
RSEM	Li et al. 2011^44^	https://github.com/deweylab/RSEM
EdgeR	Robinson et al.^45^	https://bioconductor.org/packages/edgeR/
SqueezeMeta	Tamames et al.^38^	https://github.com/jtamames/SqueezeMeta
GenBank	Clark et al.^47^	https://www.ncbi.nlm.nih.gov/genbank/
KEGG	Kanehisa and Goto^48^	https://www.genome.jp/kegg/

### Study design

Over a 24-month period, we prospectively enrolle consecutive patients aged over 18 years who presented to the Liverpool Hospital High Risk Foot Service or Emergency Department with an infected diabetic foot ulcer (DFU) occurring below the malleolus. Infections were diagnosed based on clinical observations as defined by the International Working Group for the Diabetic Foot (IWGDF) guideline on the diagnosis and treatment of foot infection in persons with diabetes^3^. Infection severity was determined using the IWGDF PEDIS classification and patients were assigned accordingly (PEDIS 2—mild infection, PEDIS 3—moderate infection, and PEDIS 4—severe infection). Tissue punch biopsies obtained in the out-patient setting were collected from each DFU after debriding and cleansing the wound with NaCl 0.9%. All out-patient-based biopsies were obtained prior to the start of any antibiotic therapy, patients who had received any systemic or topical antimicrobial therapy two weeks prior to enrollment were excluded. Surgeons collected intraoperative deep tissue specimens from patients who required surgical intervention (resection, amputation, or debridement) for management of their DFI. All tissue specimens were immediately placed in to RNA*later* (Thermo Fisher Scientific, Waltham, MA, United States) stabilization solution for 24 h at 4 °C and then stored at −80 °C until processed.

Patient demographics, laboratory and clinical data were collected through patient charts and the electronic medical records for correlation against microbiome data. Clinical data and wound metrics of interest that were collected included; PEDIS infection severity, duration of DFU prior to presentation and duration of diabetes. Laboratory data were focused on the results of white blood cell count (WBC), erythrocyte sedimentation rate (ESR), and C-reactive protein (CRP), neutrophils, and glycosylated hemoglobin (HbA1c). Ethics approval for this study was granted by the South West Sydney Local Health District Research and Ethics Committee (HREC/14/LPOOL/487, SSA/14/LPOOL/489). Informed written consent was obtained from all study participants. The study methodology was designed in accord with, and our molecular surveillance data are reported in keeping with, the “Strengthening the Reporting of Molecular Epidemiology for Infectious Diseases (STROME-ID-STROBE)” statement^39^.

### Sample size

In total 39 patients with DFI were recruited over the study period. For shotgun sequencing a total of 36 from 39 patients were analsyed. Three samples failed library preparation (Patient ID 15, 16, 30) and were excluded. 14 of the 39 samples were used for RNA analysis. Due to the poor RNA integrity from infected DFU tissue specimens, only a subset of the total patient population (14/39 samples) could be used for RNA—seq analysis. The subset of infected DFU specimens have been used and published recently by Heravi et al.^40^. In this study, the raw fastq files pertaining to RNA reads were used as input to our lab workflows.

### DNA isolation and library preparation

Frozen tissue samples were homogenized in nuclease free water using a TissueRuptor II homogoniser (Qiagen, Hilden, Germany) for 10 s at medium speed. Bacterial DNA was then extracted using the Zymo HostZero microbial DNA kit (Zymo Research, Irvine, CA, USA) according to the manufacturer’s instructions, using 200 µL of previously homogenized tissue as input. Extracted DNA was quantified using Qubit Fluorometric Quantitation (Thermo Fisher Scientific, Waltham, MA, USA) and utilized for library preparation using the Illumina Nextera DNA Flex Kit (Illumina Inc, San Diego, CA, USA). Sequencing was then performed on the HiSeq 2500 platform using high output, v4 chemistry at 2 × 126 bp.

### Processing of shotgun data

Shotgun sequencing reads were processed using Bbmerge^41^ and Bbduk for the merging of paired end reads and the removal of low-quality reads, respectively. Remaining high quality reads were then used as input for the Humann2 pipeline^12^, which utilizes the ChocoPhlan database for taxonomic classification and Uniref_90 for functional annotation. Analysis of taxonomic results was then carried out using the base R package, while functional analysis was performed using LefSe with the normalized genefamily ouput from Humman2, for identifying significant features associated with each PEDIS group.

### RNA isolation and and library preparation

Total RNA was isolated from tissue specimens using the TRIzol Plus Total Transcriptome Isolation Kit as per the manufacturer’s instructions (Life Technologies, Carlsbad, CA, USA). Briefly, samples were homogenized in Trizol (Thermo Fisher Scientific, Waltham, MA, USA) using a FastPrep-24 homogenizer (MP Biomedicals, Irvine, CA, USA) with 0.1 and 0.5 mm beads. Chloroform was added, and the sample was centrifuged to isolate the RNA containing aqueous phase. Isolated RNA was washed and purified using the supplied columns and subjected to DNAse (Thermo Fisher Scientific, Waltham, MA, USA) treatment prior to library preparation. Extracted RNA was evaluated using an Agilent 2100 Bioanalyzer (Agilent Technologies, Santa Clara, CA, USA) to ensure high quality RNA was isolated. Ribodepletion and library construction were then performed using the Illumina Ribo-Zero Gold Epidemiology kit and the Illumina TruSeq kit (Illumina Inc, CA, USA), respectively. Sequencing was then carried out on the Novaseq 6000 S4 platform at 2 × 150 bp to ensure an output of >100 million reads per sample.

### Processing of metatranscriptomic data

RNA-seq generated approximately 170 million (±20 SEM) 2 × 150 bp paired end reads per sample with a mean ribosomal content of 7.98%. Reads were trimmed using TrimGalore/Cutadapt^42^ and aligned in paired-end mode to GRCh38.p12 with alternative haplotypes and unlocalised contigs removed, using STAR2.5.4b^43^. Only samples that had a human library size of >30 million reads were retained for analysis, with samples exhibiting poor mapping likely being reflective of high microbial content. Following quality filtering, the mean effective library size which mapped to the human genome was approximately 58 million (±9.5 SEM) reads per sample, providing sufficient coverage for robust host RNA profiling. Unmapped reads were retained for microbial RNA analysis.

### Annotation of metatranscriptomic datasets

For host analysis, per-gene expression levels were called at count and transcript per million (TPM) levels using RSEM (v1.3.0)^44^. Differentially expressed genes (DEGs) were identified using edgeR^45^ by fitting a multilevel ANOVA model on per-gene read counts against the PEDIS categorical response, and then testing for post-hoc contrasts for 3 vs. 2 and 4 vs. 3. Bonferroni correction was applied to each set of *p*-values, and DEGs were defined as having a family-wise error rate (FWER) of <0.05.

Microbial analysis of RNA data was completed using the SqueezeMeta pipeline (v1.0)^38^ utilizing the co-assembly option with no binning. Briefly, paired end reads were assembled i using Megahit prior to taxonomic and functional annotation using the DIAMOND sequencing aligner to the GenBank and KEGG databases, respectively. Reads of individual samples were then be mapped to assembled contigs for the estimation of taxonomic and functional abundances using Bowtie2. Relative activity plots for each DFI phenotype were then generated using R, based on the raw read counts mapping to each taxa. Raw read counts varied across samples, ranging from 5 to 100 million reads available for downstream microbial RNA profiling. Taxonomic and functional analysis was completed in parallel using a bioconductor software package for examining differential expression of replicated count data (edgeR). Read counts were normalized (LogCPM), and a principal coordinate analysis (PCA) was performed to demonstrate any variation among datasets based on their taxonomic and functional profiles.

### Quantification and statistical analysis

The R Statistical Package (R Core Development Team, 2017) was used to generate all figures and compute statistical analysis.

### Reporting summary

Further information on experimental design is available in the Nature Research Reporting Summary linked to this paper.

## Supplementary information

Supplementary Information

Supplementary Data

Reporting Summary

## Data Availability

All raw data is provided as text output in Microsoft excel as a supplementary data file. Metatranscriptome data (RNA reads) have been deposited in NCBI Sequence Read Archive (SRA)/NCBI (http://www.ncbi.nlm.nih.gov/sra) accession number PRJNA563930. Similararily, metagenome (DNA reads) data has been deposited under accession number PRJNA610303. Availability of R scripts or command lines for any of the programs used have been included in the supplementary data file, and/or can be requested from the author.
