# Supplementary material for: A multiomics approach to identify host-microbe alterations associated with infection severity in diabetic foot infections: a pilot study
Source: NPJ Biofilms Microbiomes. 2021 Mar 22;7:29. doi: 10.1038/s41522-021-00202-x (PMC7985513; doi:10.1038/s41522-021-00202-x)

Supplementary Images

Supplementary Figure 1. Shotgun sequencing PCA plot. Analysis completed using the relative abundance data from the metaphlan2 output.

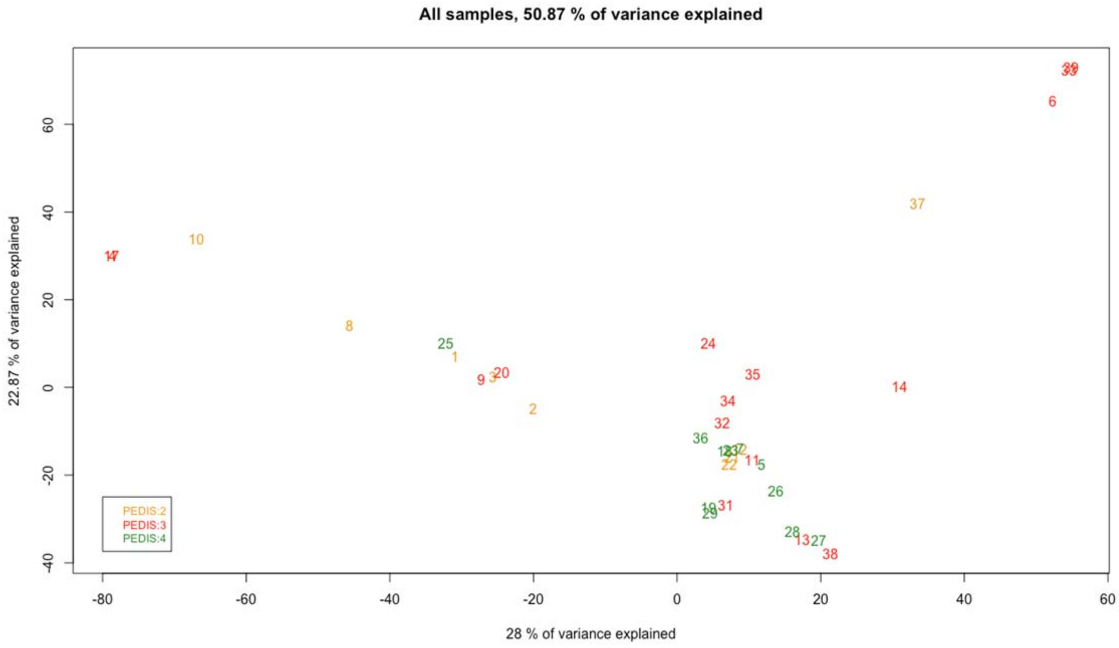

Supplementary Figure 2. Microbial transcriptome PCA plot of taxonomic activity. Analysis completed using normalised raw count data (logCPM) of taxanomic Genbank annotations.

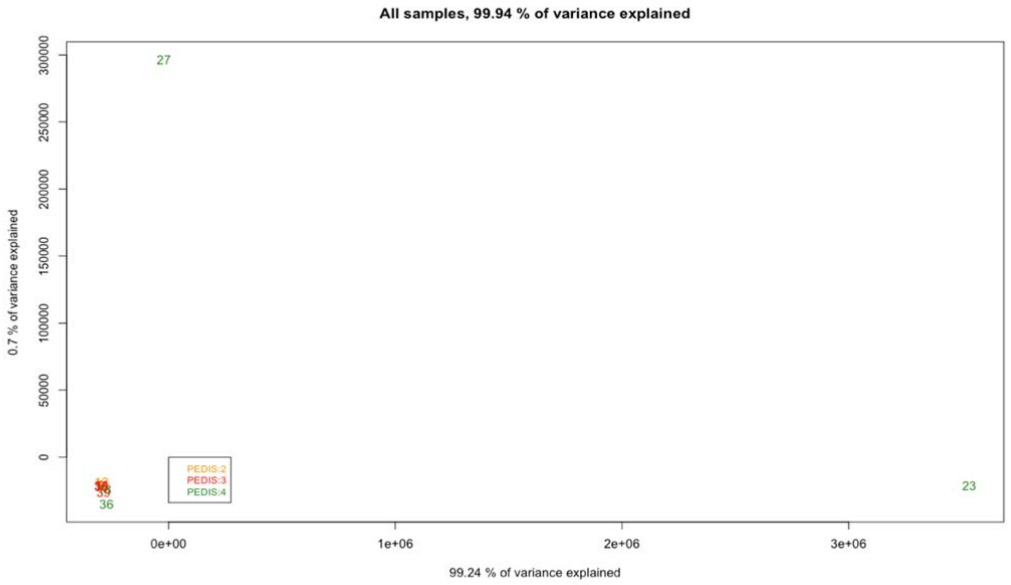

Supplementary Figure 3: Microbial transcriptome PCA plot of functional activity. Analysis completed using normalised raw count data (logCPM) of KEGG annotations.

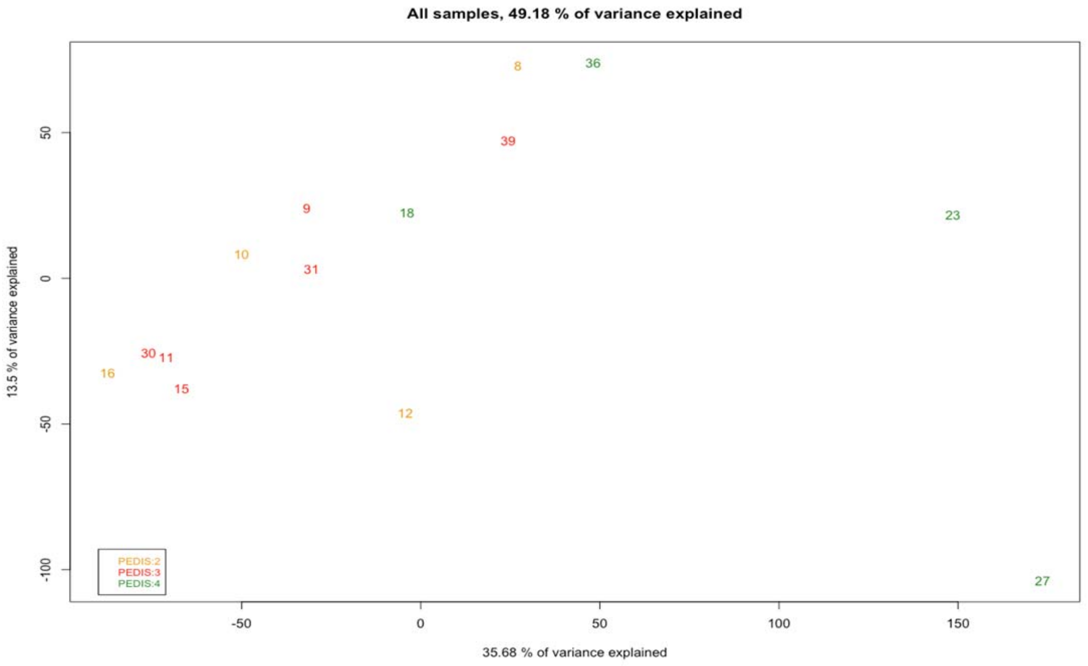

Supplementary Figure 4: Volcano plot of microbial function identifying DEGs between PEDIS 2 and 3

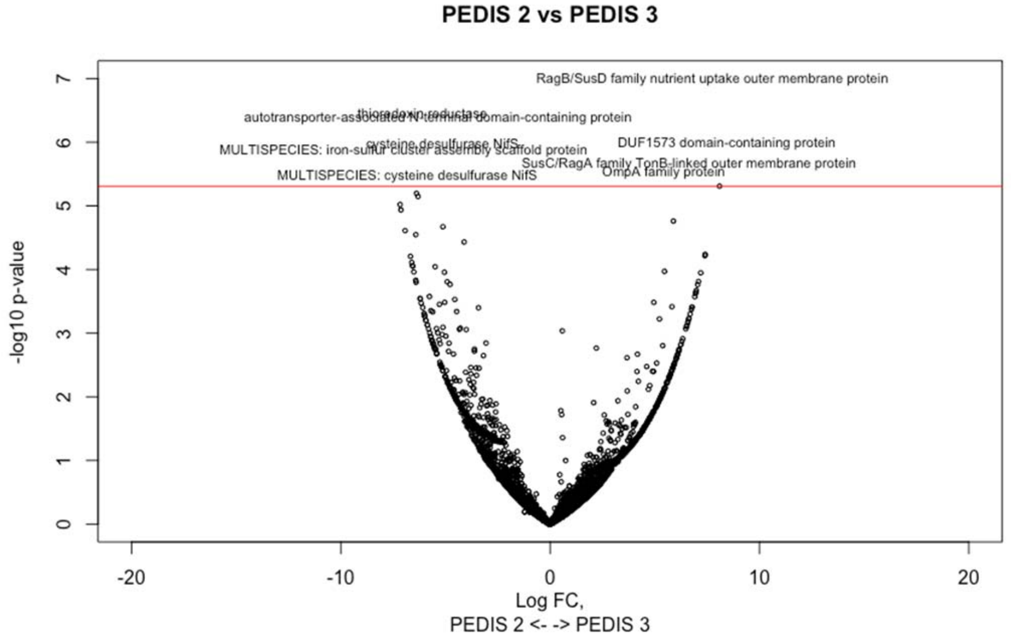

Supplementary Figure 5: Volcano plot of microbial function identifying DEGs between PEDIS 3 and 4

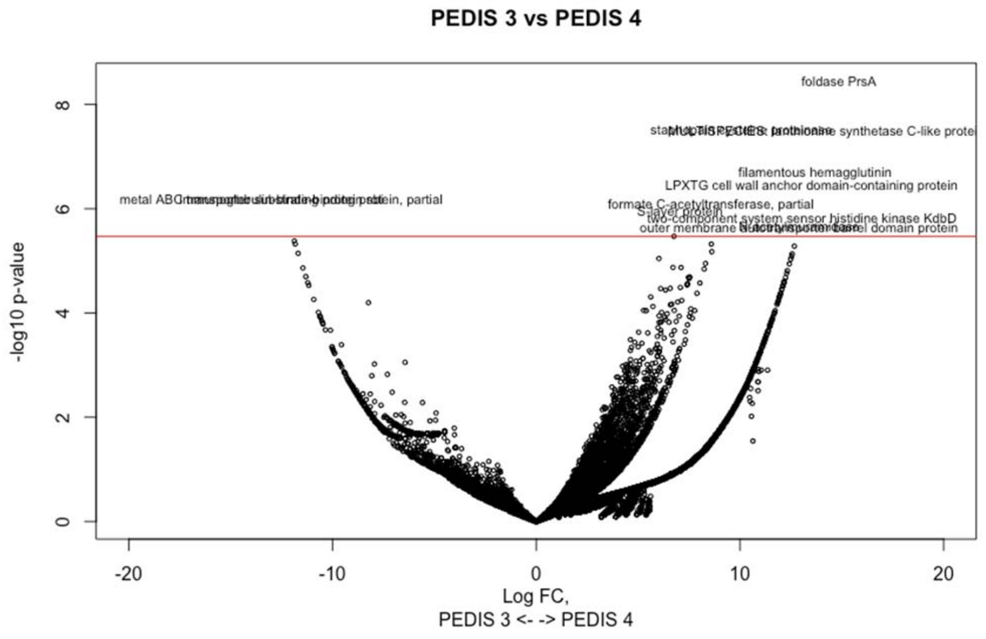

Supplementary Figure 6: Volcano plot of microbial function identifying DEGs between PEDIS 2 and 4 (SEED database)

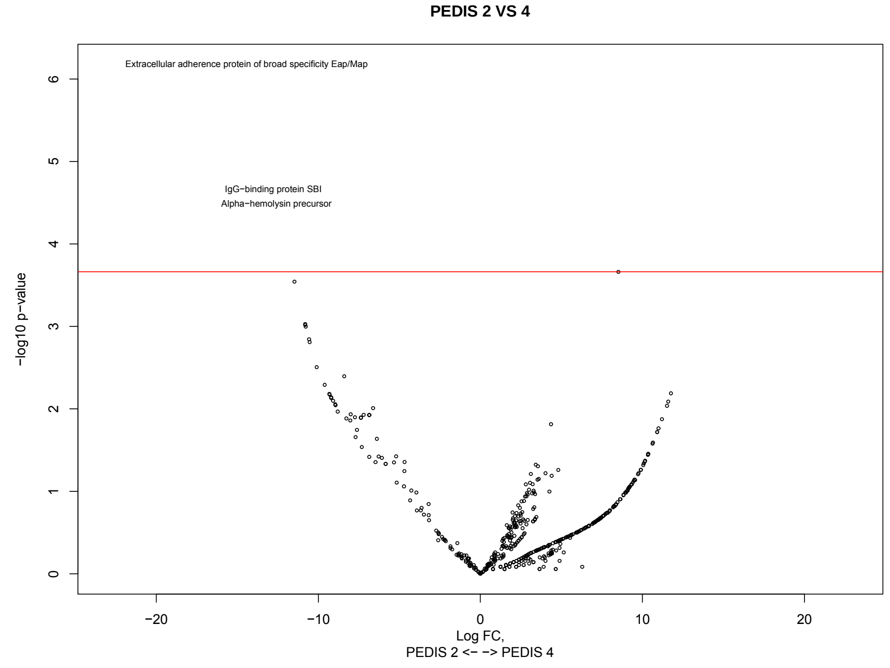

Supplementary Figure 7: Volcano plot of microbial function identifying DEGs between PEDIS 3 and 4 (SEED database)

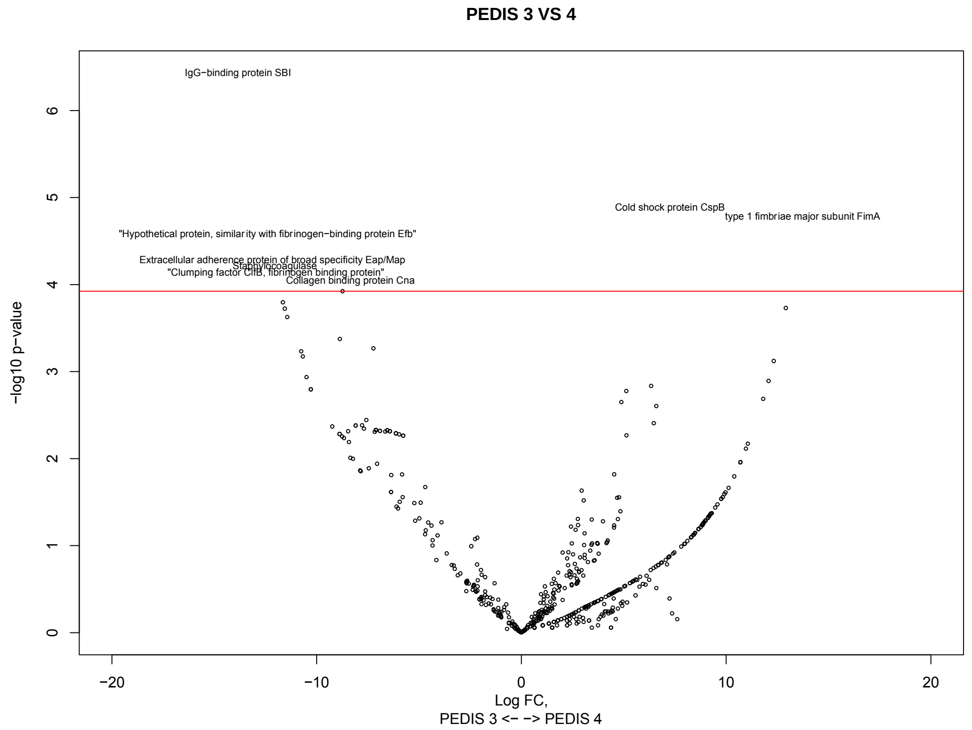

Supplementary Figure 8: Host transcriptome PCA plot

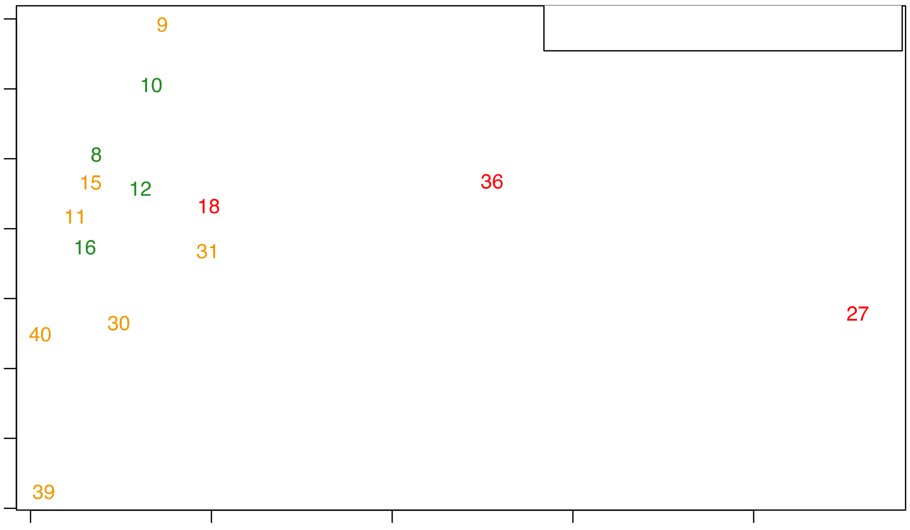

Supplementary Figure 9: Volcano plot of host DEGs between PEDIS 3 and 4 patients

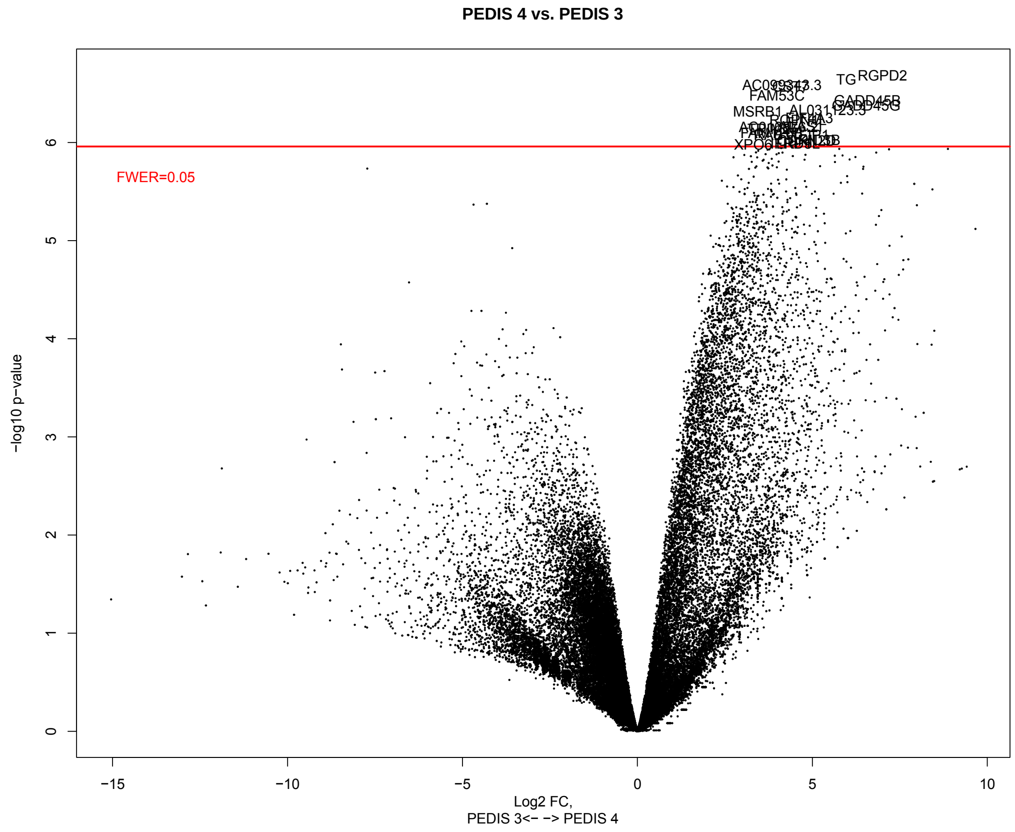

Supplementary Figure 10: Heatmap outlining alterations in gene expression relative to white cell counts.

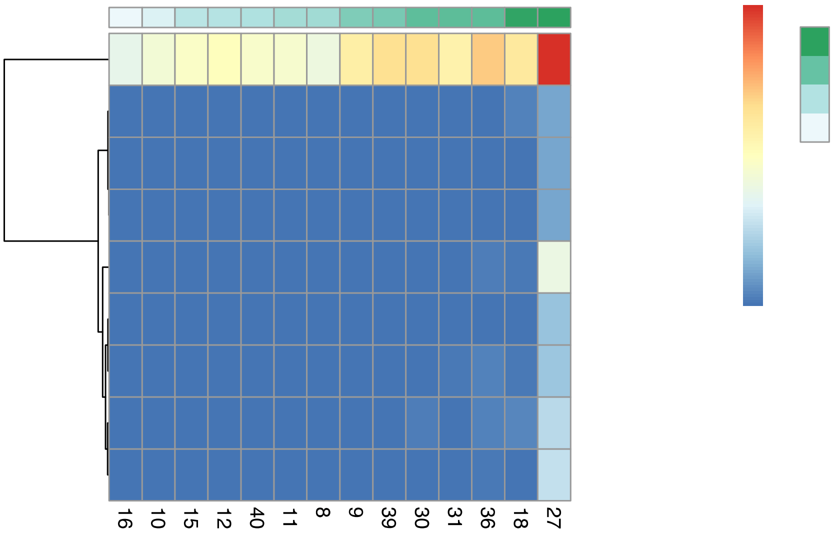

Supplement: Supplementary file 1 — Supplementary Information [file 41522_2021_202_MOESM1_ESM.pdf]
