# Supplementary material for: A multiomics approach to identify host-microbe alterations associated with infection severity in diabetic foot infections: a pilot study
Source: NPJ Biofilms Microbiomes. 2021 Mar 22;7:29. doi: 10.1038/s41522-021-00202-x (PMC7985513; doi:10.1038/s41522-021-00202-x)
Supplement: Supplementary file 3 — Reporting Summary [file 41522_2021_202_MOESM3_ESM.pdf]

nature research

Corresponding author(s): *Double-blind peer review submissions: write DBPR and your manuscript number here instead of author names.*

Last updated by author(s): *YYYY-MM-DD*

Reporting Summary

Nature Research wishes to improve the reproducibility of the work that we publish. This form provides structure for consistency and transparency in reporting. For further information on Nature Research policies, see our Editorial Policies and the Editorial Policy Checklist.

Statistics

For all statistical analyses, confirm that the following items are present in the figure legend, table legend, main text, or Methods section.

n/a	Confirmed	
☒	☐	The exact sample size (n) for each experimental group/condition, given as a discrete number and unit of measurement
☒	☐	A statement on whether measurements were taken from distinct samples or whether the same sample was measured repeatedly
☐	☒	The statistical test(s) used AND whether they are one- or two-sided<br>*Only common tests should be described solely by name; describe more complex techniques in the Methods section.*
☐	☒	A description of all covariates tested
☐	☒	A description of any assumptions or corrections, such as tests of normality and adjustment for multiple comparisons
☐	☒	A full description of the statistical parameters including central tendency (e.g. means) or other basic estimates (e.g. regression coefficient) AND variation (e.g. standard deviation) or associated estimates of uncertainty (e.g. confidence intervals)
☐	☒	For null hypothesis testing, the test statistic (e.g. F, t, r) with confidence intervals, effect sizes, degrees of freedom and P value noted<br>*Give P values as exact values whenever suitable.*
☒	☐	For Bayesian analysis, information on the choice of priors and Markov chain Monte Carlo settings
☒	☐	For hierarchical and complex designs, identification of the appropriate level for tests and full reporting of outcomes
☒	☐	Estimates of effect sizes (e.g. Cohen's d, Pearson's r), indicating how they were calculated

Our web collection on statistics for biologists contains articles on many of the points above.

Software and code

Policy information about availability of computer code

Data collection	Open source platforms used in this study which have all been cited within the methods section:<br>1. HUMAnN2 - http://huttenhower.sph.harvard.edu/humann<br>2. Bowtie2 - http://bowtie-bio.sourceforge.net/bowtie2/index.shtml<br>3. BBTools - https://sourceforge.net/projects/bbmap/<br>4. Trim Galore - https://github.com/FelixKrueger/TrimGalore<br>5. STAR - https://github.com/alexdobin/STAR<br>6. RSEM - https://github.com/deweylab/RSEM<br>7. EdgeR - https://bioconductor.org/packages/edgeR/<br>8. SqueezeMeta - https://github.com/jtamames/SqueezeMeta<br>9. GenBank - https://www.ncbi.nlm.nih.gov/genbank/<br>10. KEGG - https://www.genome.jp/kegg/
Data analysis	The R Project for Statistical Computing - https://www.r-project.org/

For manuscripts utilizing custom algorithms or software that are central to the research but not yet described in published literature, software must be made available to editors and reviewers. We strongly encourage code deposition in a community repository (e.g. GitHub). See the Nature Research guidelines for submitting code & software for further information.

Data

Policy information about availability of data

All manuscripts must include a data availability statement. This statement should provide the following information, where applicable:

- Accession codes, unique identifiers, or web links for publicly available datasets
- A list of figures that have associated raw data
- A description of any restrictions on data availability

All raw data is provided as text output in Microsoft excel as a supplementary data file. Metatranscriptome data (RNA reads) have been deposited in NCBI Sequence Read Archive (SRA)/NCBI (http://www.ncbi.nlm.nih.gov/sra) accession number PRJNA563930. Similararily, metagenome (DNA reads) data has been deposited under accession number PRJNA610303. Availability of R scripts or command lines for any of the programs used have been included in the supplementary data file, and/or can be requested from the author.

Field-specific reporting

Please select the one below that is the best fit for your research. If you are not sure, read the appropriate sections before making your selection.

☒ Life sciences ☐ Behavioural & social sciences ☐ Ecological, evolutionary & environmental sciences

For a reference copy of the document with all sections, see nature.com/documents/nr-reporting-summary-flat.pdf

Life sciences study design

All studies must disclose on these points even when the disclosure is negative.

Sample size	This was a pilot study design, and as such no sample size calculation was required. Our main goal was to achieve >20 participants which is suitable for a pilot analysis.
Data exclusions	3 patients were excluded from having both DNA and RNA analysis due to failure of library preparation - inadequate amount of DNA input. This was stated clearly in the manuscript.
Replication	N/A
Randomization	N/A
Blinding	N/A

Reporting for specific materials, systems and methods

We require information from authors about some types of materials, experimental systems and methods used in many studies. Here, indicate whether each material, system or method listed is relevant to your study. If you are not sure if a list item applies to your research, read the appropriate section before selecting a response.

Materials & experimental systems

n/a	Involved in the study	
☒	☐	Antibodies
☒	☐	Eukaryotic cell lines
☒	☐	Palaeontology and archaeology
☒	☐	Animals and other organisms
☐	☒	Human research participants
☐	☒	Clinical data
☒	☐	Dual use research of concern

Methods

n/a	Involved in the study	
☒	☐	ChIP-seq
☒	☐	Flow cytometry
☒	☐	MRI-based neuroimaging

Human research participants

Policy information about studies involving human research participants

Population characteristics	patients aged over 18 years, with diabetes mellitus and an infected diabetic foot ulcer.
Recruitment	Participants were recruited consecutively from a high risk foot clinic.
Ethics oversight	Ethics approval for this study was granted by the South West Sydney Local Health District Research and Ethics Committee (HREC/14/LPOOL/487, SSA/14/LPOOL/489).

Note that full information on the approval of the study protocol must also be provided in the manuscript.

Clinical data

Policy information about clinical studies

All manuscripts should comply with the ICMJE guidelines for publication of clinical research and a completed CONSORT checklist must be included with all submissions.

Clinical trial registration	The pilot study was not registered on a clinical trials registry
Study protocol	The full study protocol can be openly requested from the author
Data collection	The study methodology was designed in accord with, and our molecular surveillance data are reported in keeping with, the “Strengthening the Reporting of Molecular Epidemiology for Infectious Diseases (STROME-ID-STROBE)” statement. This statement was included within the manuscript. Data was collected over a 24-month period, prospectively in consecutive patients. All data was de-identified and stored on an encrypted hospital server.
Outcomes	This was an exploratory study with no -re-defined outcomes.
